# The Molecular Tumor Board of the Regina Elena National Cancer Institute: from accrual to treatment in real-world

**DOI:** 10.1186/s12967-023-04595-5

**Published:** 2023-10-16

**Authors:** Patrizio Giacomini, Fabio Valenti, Matteo Allegretti, Matteo Pallocca, Francesca De Nicola, Ludovica Ciuffreda, Maurizio Fanciulli, Stefano Scalera, Simonetta Buglioni, Elisa Melucci, Beatrice Casini, Mariantonia Carosi, Edoardo Pescarmona, Elena Giordani, Francesca Sperati, Nicoletta Jannitti, Martina Betti, Marcello Maugeri-Saccà, Fabiana Letizia Cecere, Veronica Villani, Andrea Pace, Marialuisa Appetecchia, Patrizia Vici, Antonella Savarese, Eriseld Krasniqi, Virginia Ferraresi, Michelangelo Russillo, Alessandra Fabi, Lorenza Landi, Gabriele Minuti, Federico Cappuzzo, Massimo Zeuli, Gennaro Ciliberto

**Affiliations:** 1 Clinical Trial Center, Biostatistics and Bioinformatics, IRCCS-Regina Elena National Cancer Institute, 00144 Rome, Italy; 2 UOC Translational Oncology Research, IRCCS-Regina Elena National Cancer Institute, 00144 Rome, Italy; 3 SAFU, Department of Research, Advanced Diagnostics, and Technological Innovation, IRCCS-Regina Elena National Cancer Institute, 00144 Rome, Italy; 4 Department of Pathology, IRCCS-Regina Elena National Cancer Institute, 00144 Rome, Italy; 5 Clinical Trial Center, Biostatistics and Bioinformatics, San Gallicano Dermatological Institute IRCCS, 00144 Rome, Italy; 6 Pharmacy Unit, Medical Direction, IRCCS-Regina Elena National Cancer Institute and San Gallicano Institute, 00144 Rome, Italy; 7 Medical Oncology 2, IRCCS-Regina Elena National Cancer Institute, 00144 Rome, Italy; 8 Neuro-Oncology Unit, IRCCS-Regina Elena National Cancer Institute, 00144 Rome, Italy; 9 Oncological Endocrinology Unit, IRCCS-Regina Elena National Cancer Institute, 00144 Rome, Italy; 10 Phase IV Studies, IRCCS-Regina Elena National Cancer Institute, 00144 Rome, Italy; 11 Medical Oncology 1, IRCCS-Regina Elena National Cancer Institute, 00144 Rome, Italy; 12 Sarcomas and Rare Tumors Departmental Unit, IRCCS-Regina Elena National Cancer Institute, 00144 Rome, Italy; 13 Precision Medicine Unit in Senology, Fondazione Policlinico Universitario A. Gemelli IRCCS, 00168 Rome, Italy; 14 Clinical Trial Center: Phase 1 and Precision Medicine, IRCCS-Regina Elena National Cancer Institute, 00144 Rome, Italy; 15 Scientific Direction, IRCCS-Regina Elena National Cancer Institute, 00144 Rome, Italy

**Keywords:** Molecular tumor board, Tumor DNA (tDNA), Circulating tumor DNA (ctDNA), Next generation sequencing (NGS), Digital PCR (dPCR), Target therapy, Off-label, Access to treatment

## Abstract

**Background:**

Molecular Tumor Boards (MTB) operating in real-world have generated limited consensus on good practices for accrual, actionable alteration mapping, and outcome metrics. These topics are addressed herein in 124 MTB patients, all real-world accrued at progression, and lacking approved therapy options.

**Methods:**

Actionable genomic alterations identified by tumor DNA (tDNA) and circulating tumor DNA (ctDNA) profiling were mapped by customized OncoKB criteria to reflect diagnostic/therapeutic indications as approved in Europe. Alterations were considered non-SoC when mapped at either OncoKB level 3, regardless of tDNA/ctDNA origin, or at OncoKB levels 1/2, provided they were undetectable in matched tDNA, and had not been exploited in previous therapy lines.

**Results:**

Altogether, actionable alterations were detected in 54/124 (43.5%) MTB patients, but only in 39 cases (31%) were these alterations (25 from tDNA, 14 from ctDNA) actionable/unexploited, e.g. they had not resulted in the assignment of pre-MTB treatments. Interestingly, actionable and actionable/unexploited alterations both decreased (37.5% and 22.7% respectively) in a subset of 88 MTB patients profiled by tDNA-only, but increased considerably (77.7% and 66.7%) in 18 distinct patients undergoing combined tDNA/ctDNA testing, approaching the potential treatment opportunities (76.9%) in 147 treatment-naïve patients undergoing routine tDNA profiling for the first time. Non-SoC therapy was MTB-recommended to all 39 patients with actionable/unexploited alterations, but only 22 (56%) accessed the applicable drug, mainly due to clinical deterioration, lengthy drug-gathering procedures, and geographical distance from recruiting clinical trials. Partial response and stable disease were recorded in 8 and 7 of 19 evaluable patients, respectively. The time to progression (TTP) ratio (MTB-recommended treatment vs last pre-MTB treatment) exceeded the conventional Von Hoff 1.3 cut-off in 9/19 cases, high absolute TTP and Von Hoff values coinciding in 3 cases. Retrospectively, 8 patients receiving post-MTB treatment(s) as per physician’s choice were noted to have a much longer overall survival from MTB accrual than 11 patients who had received no further treatment (35.09 vs 6.67 months, p = 0.006).

**Conclusions:**

MTB-recommended/non-SoC treatments are effective, including those assigned by ctDNA-only alterations. However, real-world MTBs may inadvertently recruit patients electively susceptible to diverse and/or multiple treatments.

**Supplementary Information:**

The online version contains supplementary material available at 10.1186/s12967-023-04595-5.

## Background

Molecular Tumor Boards (MTB) are multidisciplinary panels assigning targeted therapies based on genomic tumor profiling, mostly by tumor-agnostic (histology-independent) criteria. They have been the subject of two systematic reviews. The former took into account 40 reports during the previous 10 years for a total of 6303 patients, of whom 1107 (17.6%) received MTB recommendations for treatment [1]. The latter focused on 14 studies reporting the outcomes of 3328 patients treated as per MTB recommendations. Response rates were as variable as 0–67%, and only 1/14 studies compared the efficacy of MTB-recommended treatments vs physician’s choice [2]. These figures reveal the pervasive role of MTBs in precision oncology, but at the same time highlight profound differences in case mix, accrual/eligibility criteria, genomic profiling, alteration mapping, therapy assignment, and non-technical (e.g. reimbursement) support measures.

Despite two identical MTBs are hard to find, two major types of MTB may be tentatively distinguished: those operating in real-world, and those with steering prerogatives, acting in the context of structured precision oncology trials. Real-world MTBs operate with non-consensus patient selection criteria [3, 4]. They consider advanced cancer patients with no residual therapeutic opportunity, e.g. the patient population most frequently seen in clinical practice. Unfortunately, their complex case mix makes it difficult to define a control arm, and hence assess therapeutic efficacy in real-world [5–7]. In contrast, MTBs with a dedicated steering function on clinical trials have their focus on a pre-selected set of specific drug/alteration matches. Steering MTBs were able to apply rigorous case–control and randomization approaches to estimate therapeutic efficacy [8–11], and linked actionability levels to outcome [12], but at the cost of introducing some selection bias in the patient population.

Active since September 2018, the MTB of the Regina Elena Institute, Rome, Italy (IRE-MTB) has been established as a typical real-world, tumor-agnostic MTB. As per statutory aim, the IRE-MTB focuses on actionable alterations in patients with advanced cancer and no Standard of Care (SoC) therapeutic opportunities. Herein, we report on our own pre-set accrual criteria, distinctive operational flowchart, as well as customized definitions of alteration mapping criteria for tissue and circulating actionable alterations. Based on these definitions, we measured outcome (the major technical limitation of real-world MTBs), by applying several metrics. Favorable responses to MTB-recommended treatments were noted in a distinguishable subset of patients. This bears implications on the nature and ultimate goal of real-world MTBs.

## Methods

### Patients

Two distinct cohorts of patients were considered: MTB patients strictly speaking (n = 124) with progressive disease and no approved therapy option, and patients routinely NGS profiled (n = 147) for standard therapy assignment as recommended by the European Society of Medical Oncology (ESMO) [13]. MTB patients were recruited and individually discussed by the MTB between September 1, 2018 and July 31, 2022 (dates of MTB presentation). Follow up is updated for all patients to January 31, 2023. Enrolment criteria were as follows: (a) any neoplastic condition, (b) > 18 yr, (c) no SoC treatment options, (d) available tumor tissue and/or circulating tumor DNA (tDNA and/or ctDNA), and (e) Performance Status (PS) ≤ 2. There were no exclusion criteria except refusal to sign an informed consent. Routine Next Generation Sequencing (NGS) patients were enrolled in the context of clinical genotyping (August 1, 2020 to July 31, 2022; dates of NGS diagnostic report).

### Study design and genomic alteration mapping criteria

Enrolment was prospective. Testing was real-time. Analysis was retrospective. The primary study aim was to enumerate non-standard-of-care (non-SoC) actionable alterations in the MTB setting. The secondary aim was to assess the outcome of MTB-recommended treatment. Genomic alterations were primarily classified by the OncoKB scale, complemented by the ESCAT-ESMO scale [14, 15] through a commercial decision support tool (Oncomine Knowledgebase Reporter; OKR, Life Technologies). However, lacking an online ESCAT-informed knowledgebase, the OncoKB scale was adapted to reflect approval (at the time of MTB accrual) by the European and Italian Medicinal Agencies (EMA and AIFA), that usually lag behind Food and Drug Administration (FDA). MTB patients were considered potentially eligible for non-standard therapy when their tumor alteration(s) met either or both the IRE-MTB mapping criteria: (a) OncoKB levels 3A/B in tDNA and/or ctDNA at the time of MTB accrual, (b) OncoKB levels 1/2 in ctDNA, as long as not previously detected in tDNA, and not previously exploited for target therapy.

Testing was hierarchical: tDNA first, and then ctDNA. Accordingly, the MTB resorted to ctDNA exclusively when tDNA was either not available or not suitable. tDNA was not suitable when obtained prior to the two most recent lines of therapy, and/or > 12 months before MTB accrual. Selected patients were ctDNA-tested to orthogonally validate specific alterations and/or longitudinally monitor their blood clearance during targeted treatment.

### Molecular profiling

Pre-analytical processing and targeted NGS were carried out on DNA from tumor tissue (tDNA) and/or circulating tumor DNA (ctDNA) as described [16]. For tDNA, targeted NGS was carried out by the Oncomine Focus Assay (OFA), Oncomine Comprehensive Assay plus (OCA Plus), Ion Ampliseq Cancer Hotspot panel v2 (CHPv2), all from Thermofisher, and FusionPlex Solid Tumor and Sarcoma panels (Fusionplex) from Archer. Whole Exome tDNA Sequencing (WES) of selected cases was performed using the SureSelectXT low input Reagent kit and the Clinical Research Exome kit (Agilent). Whole Transcriptome Sequencing (WTS) was performed with the Illumina Stranded Total RNA Prep with the Ribo-Zero Plus kit (Illumina), and analyzed with Sarek version 3.1 (https://nf-co.re/sarek) to detect germ line and somatic variants. Genes and variants were annotated with VEP (Variant Effect Predictor). Oncogenic and clinically actionable mutations were identified with PCGR (Personal Cancer Genome Reporter). RNA-seq data were analyzed by the Kallisto v0.46.2 18, and Sleuth v0.30.019 recommended pipelines. ctDNA was sequenced by the Thermofisher Oncomine Pan-Cancer Cell-free assay (Pan-Cancer). Alternatively, tDNA and ctDNA sequencing was outsourced (Foundation One CDx, Foundation One CDx, and Foundation One Liquid). Reverse Transcription PCR (RT-PCR) and dgital PCR (dPCR) were carried out on either or both tDNA and ctDNA using the Thermofisher QuantStudio 3D and custom-designed assays, as described [16].

### Annotation and recording

Patients with germ line variants were referred to our genetic counseling unit. The IRE MTB focused on patients with actionable somatic alterations (referred to as actionable alterations hitherto) from the MTB and routine NGS groups. These were recorded in a dedicated webapp, called Molecular Tumor Board Orchestrator (MTBO), that will be the subject of a separate report. Briefly, it was developed (frontend and backend) in Visual Basic with an SQL server database handling/recording meeting calls, email announcements, molecular testing/annotation, outcomes, and the final MTB Report.

### Biostatistics

Categorical variables were reported as absolute and relative frequencies. Continuous variables were reported as means, standard deviations (SD), median, 95% confidence intervals (95%CI), and min–max intervals. The Kolmogorov–Smirnov normality test was applied to continuous variables. Associations between categorical variables were assessed by the χ^2^ test of independence. Outcome of MTB treatment was calculated according to Von Hoff [17], e.g. the Time to Progression (TTP) ratio was calculated (MTB-recommended therapy vs last pre-MTB standard therapy line) in each patient. A univariate logistic regression model was used to identify variables impacting the Von-Hoff ratio (≥ 1.3 vs < 1.3). The Mann–Whitney test was used to compare post-MTB treatment vs previous treatments and number of previous lines of therapy. The Kaplan–Meier product-limit method and the log-rank test were used to estimate and compare survival curves. A p-value < 0.05 was considered statistically significant. All elaborations were carried out by SPSS v. 21 (SPSS Inc., Chicago IL, USA).

## Results

### MTB workflow and attendance model

**Fig. 1:**
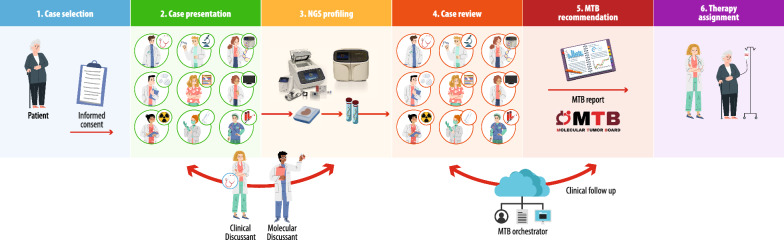
The six-step IRE MTB workflow. Step 1: informed consent. Step 2: case presentation during the first round of collegial discussion. Step 3: genomic profiling, mostly involving NGS on tDNA and ctDNA, either de novo or as a complement to the available genomic profiling. Step 4: joint appointment of two case discussants, providing distinct pieces of expertise, clinical and molecular. Step 5: case review in a second discussion round (typically 15 days later) and MTB recommendations. Step 6: treatment recommendation. Therapy is ultimately decided by the oncologist (usually the clinical discussant) and agreed with the patient

The IRE MTB operational workflow involves 6 steps, from informed consent to therapy assignment (Fig. 1) with a strictly enforced turnaround time of 15 days from case presentation to recommended therapy (e.g. between the 2 discussion rounds, step 2 to step 5). The entire workflow and patient outcomes are real-time annotated and traceable by the dedicated MTBO webapp. Active members and main attendants (Fig. 1, step 2) include oncologists, molecular biologists/biotechnologists, pathologists, biostatisticians and bioinformaticians, a hospital pharmacist, a delegate from the Institutional Biobank, the Scientific Secretary and an executive Secretary. Hematologists, endocrinologist-oncologists, a medical geneticist, interventional radiologists and radiotherapists, surgeons, patient advocacy delegates, nurses, data managers and representatives of the local health governance are invited to participate depending on the topics being addressed, as per the MTB agenda distributed at least 24 h in advance. Two discussants (clinical and molecular) are appointed for each MTB case. Most patients are recruited with a ‘packet’ of previously gathered diagnostic information, sometimes including NGS. This is considered during the first discussion round. The MTB would then request additional NGS for the same or additional specimens, and/or a variety of alternative molecular profiling tests. This extended information packet is considered in the second discussion round.

### MTB molecular profiling vs routine NGS

**Fig. 2:**
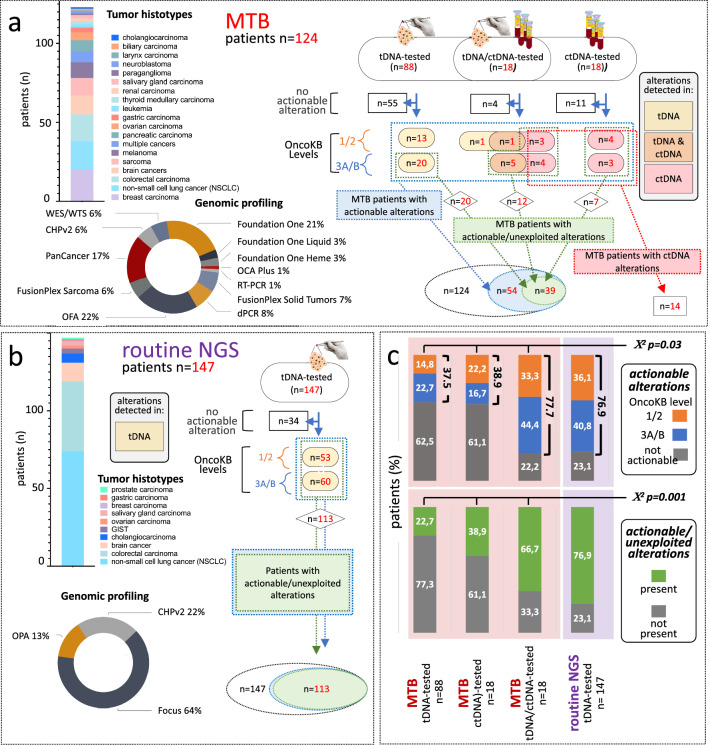
MTB and routine NGS. **a** Bar plot showing MTB case mix (tumor types), pie chart displaying NGS and other profiling approaches (for abbreviations see the specific section), and flow diagram grouping patients by testing: tDNA, ctDNA and tDNA/ctDNA. For each group, number of patients are displayed without and with actionable alterations, sorted by OncoKB level and color-coded by origin (from tDNA, ctDNA or both: yellow, red and orange, respectively). Dotted blue, green and red boxes identify patients with actionable alterations of any kind, with actionable/unexploited alterations in the MTB setting, and alterations exclusively detected in ctDNA, respectively. **b** case mix, molecular profiling and flow diagram (tDNA only) from patients undergoing routine NGS. **c** actionable and actionable/unexploited alterations (top and bottom histograms, respectively), and their OncoKB level distribution among the three MTB groups and routine NGS patients. Group significance is noted

To highlight MTB-distinctive features, real-world MTB profiling of heavily pre-treated patients was compared to in-house routine NGS profiling of treatment-naïve patients. Compared to routine NGS, the IRE MTB examined a much wider spectrum of neoplasms including rare tumors with poorly actionable genomes, and deployed a richer portfolio of tDNA molecular tests, such as custom-made and outsourced NGS panels from commercial vendors, WES, RNA-seq, and RT-PCR, as well as specific NGS and dPCR approaches for ctDNA testing (Fig. 2a vs b, bar histogram and donut charts).

Testing results of MTB and routine NGS cases were arranged by tumor type, and the prevalence of actionable alterations was compared between the two populations (Additional file 1: Table S1a vs S1b). Three distinctive features of the MTB setting were evident: (a) under-representation of cancers rich in genomic alterations such as NSCLC (18/124 vs 75/147, e.g. 14.5% vs 51%); (b) under-representation of actionable alterations in any given tumor type (e.g. NSCLC: 77.8% vs 93.3%); and (c) under-representation of actionable alterations in the setting-specific alteration pool (e.g. NSCLC: 29.2% vs 61.9%). Under-representation was significant, as assessed in a subset of tumors common to the MTB and routine NGS settings (Additional file 1: Table S1c, χ^2^ p < 0,001). Remarkably, under-representation was noted despite more intensive profiling in the MTB setting. In summary, MTB cases were depleted in actionable alterations mainly due to the specific case mix, but counter-selection at advanced disease stages in heavily pre-treated patients may have contributed to the observed unbalance, outlining multiple challenges in MTB profiling.

### tDNA and ctDNA testing

Hierarchical testing (tDNA-first, and then ctDNA in selected patients) sorted MTB patients into 3 groups profiled by tDNA (n = 88), ctDNA (n = 18), and combined tDNA/ctDNA testing (n = 18), respectively (Fig. 2, flow diagram). The flow diagram also displays case numbers from each group, absence and presence of actionable alterations, as well as their OncoKB levels and origin (from tDNA, ctDNA or both; color-coded).

Altogether, 54 MTB patients carried at least one actionable tDNA/ctDNA alteration, but due to heavy pre-treatment only 39 of them carried alterations not yet exploited at the time of MTB accrual (Fig. 2a; actionable vs actionable/unexploited alterations, blue-dotted vs green-dotted boxes, summary in Venn diagram). Interestingly, of 39 cases with actionable/unexploited alterations, 25 were identified by tDNA testing (5 of which confirmed by ctDNA), and 14 were exclusively identified through ctDNA testing (Fig. 2a, green-dotted vs red-dotted contours). Identification in ctDNA but not tDNA was due either to a lack of matched suitable (see Methods) tDNA (7 cases) or, more interestingly, to serendipitous discovery of novel ctDNA-only actionable alterations (the remaining 7 cases). In these 7 patients, ctDNA testing had been requested by the MTB just to confirm the presence of alterations detected in tDNA 30 to 361 (median 146) days before. Discovery of ctDNA-only mutations and rearrangements was not artifactual because (Additional file 1: Table S2) mutational hits were within coverage of tDNA-grade and ctDNA-grade NGS panels adopted by the MTB. Moreover, three ctDNA-only SNVs (pts 15, 24, and 39) could be orthogonally confirmed by dPCR in both tDNA and ctDNA.

At variance from MTB testing, routine NGS profiling was carried out exclusively on tDNA, resulting in a much simpler flow diagram (Fig. 2b). Given the treatment-naïve setting, actionable alterations (n = 113) were all unexploited (Fig. 2b, overlapping blue- and green-dotted boxes and Venn diagram).

For synoptic comparison, two histograms were generated, separately showing the distribution of alterations (actionable vs actionable/unexploited) among the three MTB groups and the routine NGS group (Fig. 2c). MTB patients with actionable alterations were significantly less numerous in the tDNA and ctDNA single-testing groups than in the tDNA/ctDNA double-testing group (37.5% and 38.9% vs 77.7%; χ^2^ p < 0.03). Accordingly, patients with actionable/unexploited alterations progressively increased in the tDNA, ctDNA and tDNA/ctDNA groups (22.7% vs 38.9% vs 66.7%; χ^2^ p < 0.001), each group being significantly different from the others. In summary, whichever the alteration metric (actionable or actionable/unexploited), combined tDNA/ctDNA testing outperformed single-testing.

Due to obvious selection bias (differences in recruitment, testing, and alteration mapping criteria) MTB and routine NGS groups could not be rigorously compared. Nevertheless, patients with alterations (actionable and actionable/unexploited) drastically differed between the two tDNA-tested groups (MTB vs routine NGS: 37.5% vs 76.9%, and 22.7% vs 76.9%, respectively), whereas the tDNA/ctDNA MTB group and the routine tDNA NGS group were similar (77.7% vs 76.9%, and 66.7% vs 76.9%). These results further highlight the MTB diagnostic challenge, and show how ctDNA testing may mitigate this challenge.

### Downstream patient tracking: from routine NGS to MTB accrual

**Fig. 3:**
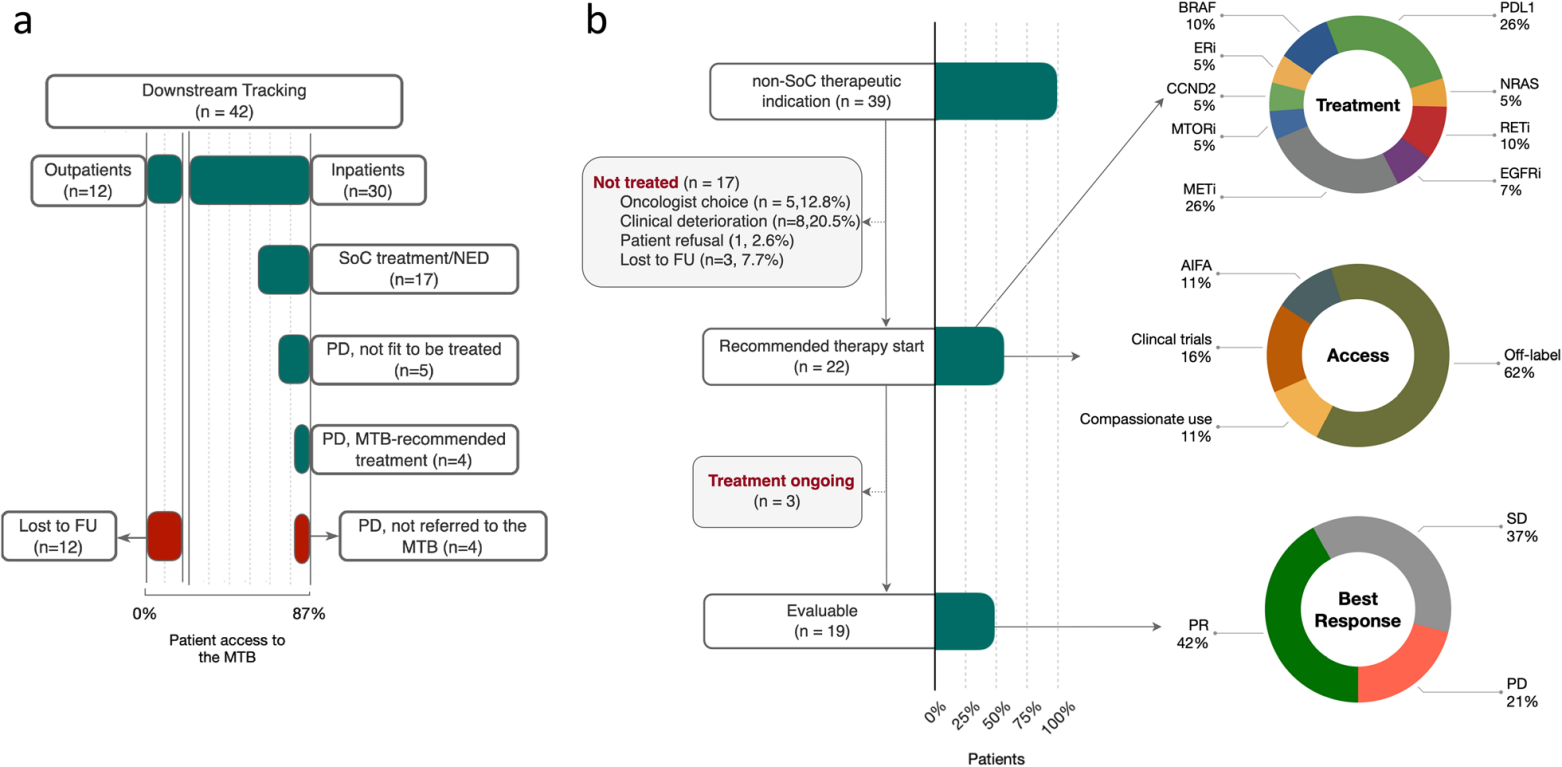
Patient journey and access to treatment. Flow diagrams showing, top to bottom: (**a**) downstream tracking of patients from routine NGS to MTB, and (**b**) drop-off (and reasons thereof) from MTB accrual to evaluation. Classes of therapeutic agents, access to therapy, and best clinical responses are noted (donut charts, right)

All MTB patients were individually discussed, whereas routine NGS cases were considered in aggregate, mainly to determine whether OncoKB 3A/B alterations, incidentally discovered at the time of routine NGS, might be reconsidered by the MTB in the event of clinical progression, for possible non-SoC therapy assignment. Unfortunately, only a subset of 42/147 patients (12 outpatients and 30 inpatients) could be tracked downstream of routine genomic profiling (Fig. 3a). These were all patients with gastrointestinal tumors, e.g. colorectal (n = 26) and stomach (n = 9) carcinomas, cholangiocarcinomas (n = 6), and a pancreas adenocarcinoma. Of interest, whereas all 12 outpatients were lost to follow-up, all the 30 inpatients were successfully tracked. Of these, 17 were still free of disease or on SoC treatment at censoring (July 31, 2022). Five had progressed and, although potentially eligible for non-SoC treatment, were not reconsidered by the MTB because not sufficiently fit. Four were reconsidered and MTB-recommended for therapy, that was followed in 3 cases and declined by one patient. Only 4 potentially eligible inpatients failed to be referred to the MTB. In summary, inpatients and outpatients could be downstream tracked in 26/30 and 0/12 cases (87% vs 0%) respectively, e.g. in our experience outpatients had no opportunity to access MTB expert opinion.

### From recommendation to treatment

All the 39/124 (31%) MTB patients with actionable/unexploited alterations (Fig. 2a, green-dotted boxes) were recommended for treatment by the MTB, but only 22/39 (56% approximately) received MTB-recommended treatment (Fig. 3b, flow diagram), for several reasons. The oncologist’s decision not to treat was based on available non-target therapies or patient-specific contraindications, whereas drop-off was mainly due to objective clinical deterioration (ECOG PS > 2 at the time of NGS report), often combined with lengthy drug-gathering procedures and/or geographical distance of centers hosting clinical trials. Only one patient refused MTB-recommended therapy, but later she elected to be treated as per MTB recommendation at another Comprehensive Cancer Center in the Rome area. Three patients initially agreed to be treated, but left Italy for personal reasons. One of them informed us that he was on treatment in North America as per MTB recommendation. These 3 patients were considered lost to follow up. No patient withdrew after receiving treatment.

Of 22 treated patients, 19 were monitored until progression/toxicity of the last administered treatment, either MTB-recommended or post-MTB (physician’s choice, see below), e.g. they were considered evaluable for response and toxicity (3 patients too early). Two of 19 patients experienced toxicity (G3) during MTB-recommended treatment.

Targeted agents were administered to 18/19 patients on the basis of single-gene alterations. The remaining case was treated by immune checkpoint blockade based on high mutation burden (Fig. 3b, top donut chart). Treatment was most often secured by public money, e.g. institutional funds (collectively defined off-label), and special funds by the Italian Medicinal Agency (AIFA 5% funds). Sponsored and non-profit clinical trials available at our institution or in the Rome area, and compassionate use were much less frequent (Fig. 3b, middle donut chart). Partial response and stable disease were by far the most frequent objective responses of MTB-recommended therapy (Fig. 3b, bottom donut chart).

### Clinical cases

**Fig. 4:**
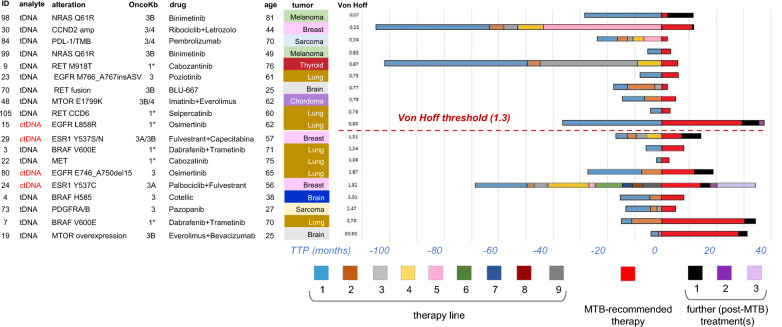
MTB clinical cases: features and outcomes. Patients treated as per MTB recommendation are ranked top to bottom by the Von Hoff TTP ratio. Main features are shown. ctDNA-tested cases are highlighted in red. TTP of pre-MTB therapy lines (color-coded) are shown as bar histograms. TTP of MTB-recommended treatment (red bars) and further (post-MTB) treatment(s) are also color-coded. Asterisks: present OncoKB level noted, OncoKB levels 3A/B on accrual. *TTP* time to progression

The 19 evaluable MTB cases are individually displayed in Fig. 4, that summarizes the considerable variability with regard to demographics, clinical-pathological and molecular features, as well as number (n = 1–9, median 2) and duration (bar graphs, 1.94–105.13 months, median 15.28) of pre-MTB therapy lines. Bar graphs also show TTP for both MTB-recommended treatment and further post-MTB treatment (one to three lines), the latter administered to 8/19 patients as per physician’s choice and solely on the basis of sufficient fitness. Since residual molecular targets had all been exploited in previous pre-MTB and MTB-recommended therapy lines, chemotherapeutic agents were assigned in indication for the specific clinical condition. The MTB did not express any recommendation at this post-MTB stage. Remarkably, TTP was similar in the MTB and post-MTB settings (95% CI 2.03–13.87 months, median 7.95 vs 95% CI 2.10 to 22.77 months, median 8.51), suggesting continued susceptibility of these patients/tumors.

Finally, Fig. 4 shows that 6 patients may be considered borderline (SoC/non-SoC), because tDNA-assigned treatment was non-SoC (Onco KB levels 3A/B) at the time of MTB accrual, but is now in indication (OncoKB levels 1/2, asterisk). In one case (pt n. 15) non-SoC treatment was assigned by non-SoC diagnostics, e.g. a ctDNA-only alteration.

### Outcome of MTB-recommended treatments

Two outcome metrics were recorded: TTP and Von Hoff ratio [17], the latter calculated by dividing the TTP of MTB-recommended therapy by the TTP of the last pre-MTB therapy line. Conventionally, a ≥ 1.3 TTP ratio marks plausible clinical efficacy [17]. Ranking MTB patients by increasing (top to bottom) Von Hoff ratios shows that 9/19 patients exceed this conventional cut-off (Fig. 4, below the red line). Remarkably, 2/3 patients (n. 7 and 19) with the longest TTP (> 24 months) were the extreme bottom outliers in the high Von Hoff range, and a third patient (n. 15) was close to cut-off. Likewise, patients n. 15, 29, 80 and 24 (all assigned non-SoC treatment by ctDNA) were in the high brackets of either or both TTP and von Hoff metrics. Altogether, these findings suggest that population-specific and patient-specific outcome metrics concordantly identify clinical benefit of tDNA and ctDNA therapy assignments.

### Variables associated with the outcome of MTB-recommended treatments

**Fig. 5:**
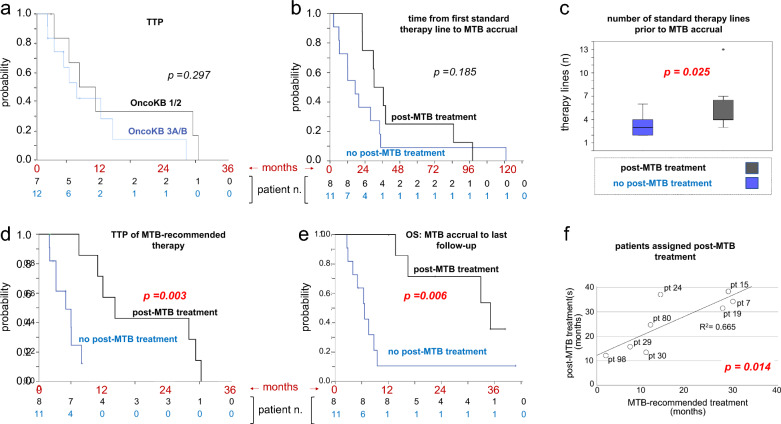
Outcome of MTB-recommended treatment. (**a)** Kaplan–Meier analysis of TTP by OncoKB level (all 19 patients treated as per MTB recommendations). (**b**–**e)** Comparisons of MTB patients receiving further post-MTB treatment(s) vs patients receiving no further treatment (n = 8 vs 11, black vs blue). Variables selected for comparison: (**b**) time from first standard therapy line to MTB accrual, (**c**) number of pre-MTB therapy lines, (**d**) TTP of MTB-recommended therapy, and (**e**) Overall Survival (OS) from MTB accrual to last follow up. (**f)** linear regression of treatment duration (MTB-recommended vs post-MTB treatments) for each of the 8 patients receiving post-MTB treatment(s). Patient ID numbers are from Fig. 4. When significant, differences/correlations are noted in red.

Several metrics were explored with no success. Von Hoff ratios > 1.3 were not significantly associated with age (Odd ratio, OR = 0.98, p = 0.507), gender (OR = 1.33, p = 0.764), best objective response to MTB-recommended therapy (OR = 0.29, p = 0.330), number of previous therapy lines (OR = 1.13, p = 0.601), or time from diagnosis to MTB referral (OR = 0.99, p = 0.428) in the overall population of 19 evaluable MTB patients. Likewise, we were unable to establish significant correlations between OncoKB level (1/2 or 3A/B) and either objective response rate or TTP of MTB-assigned treatment (Fig. 5a).

Then, it was noted that the 8 patients receiving post-MTB treatment(s) had a more favorable profile compared with the 11 patients who had received no further treatment, in that they displayed: a longer duration (non-significant trend, Fig. 5b) and a greater number (p = 0.025, Fig. 5c) of standard therapy lines prior to MTB referral, a longer TTP of MTB-recommended therapy (14.23 vs 5.03 months, CI 95% 8.49–19.97 vs 1.32–8.73, p = 0.003, Fig. 5d), and a much longer overall survival calculated from the time of MTB accrual (35.09 vs 6.67 months, CI 95% 14.85–55.32 vs 4.35–9.00, p = 0.006, Fig. 5e). Moreover, patient-by-patient regression analysis of the 8 post-MTB-treated patients demonstrated a linear correlation in treatment duration (MTB-recommended vs post-MTB; Fig. 5f). Altogether, Fig. 5c–e indicate that patients experiencing a greater number and variety of therapy lines and a longer TTP during their pre-MTB clinical course are likely to also experience a longer TTP during both MTB-recommended and post-MTB treatment(s), possibly resulting in a longer OS. Figure 5f shows that tumor/patient-specific factors may be involved.

## Discussion

The IRE MTB is similar to other real-world MTBs. Similarities include multidisciplinary composition, operational workflow, virtual attendance, cloud data recording, and focus on rare tumors [this report and see [1, 18]]. However, a systematic review [2] and our own recent survey of 16 Italian MTBs [18] reveal at least one surprising difference. As per statutory mission, the IRE MTB exclusively focuses on patients lacking SoC treatment opportunities. In contrast, many MTBs review both SoC and non-SoC indications. As noted [2], this confounds the definition of target populations and study aims, complicates retrospective comparisons, and hinders objective assessments of the MTB impact on clinical practice. In the absence of a general consensus on MTB eligibility and alteration mapping procedures, we were prompted to tentatively propose customized criteria.

The OncoKB scale was localized to reflect regulatory approval at the time and place of enrolment, and the limited endorsement of ctDNA diagnostics, presently restricted to a few EGFR alterations in EU. In addition, we distinguished actionable alterations (as per the OncoKB scale) from actionable/unexploited alterations (as per IRE MTB recruitment and alteration mapping criteria). The latter best reflect true residual therapeutic opportunities in extensively pre-treated patients.

Application of the IRE MTB alteration mapping criteria also shows that 6/19 evaluable MTB patients may be considered borderline, since their treatments, now approved, had been mapped at OncoKB levels 3A/B on accrual, and (in one of six cases) genomic profiled had been carried out by non-SoC ctDNA testing. Classifying borderline cases as non-SoC may be questionable, but resorting to pre-defined criteria of some kind is in our opinion unavoidable, and should be the subject of future harmonization guidelines.

### tDNA profiling in the SoC and non-SoC settings

The IRE MTB discussed (individually) 124 cases, and reviewed (in aggregate) 147 cases routinely NGS profiled as per ESMO guidelines [13]. Actionable alterations were strongly under-represented in tDNAs from the former compared to the latter (37.5% vs 76.9%), and the gap expanded (22.7% vs 76.9%) when actionable/unexploited alterations were considered. As expected, these sharply dropped in heavily pre-treated MTB patients (therapy lines n = 1–9, median 2), but not in treatment-naïve patients undergoing routine NGS.

It is acknowledged that profound differences in case mix and testing methods preclude stringent comparisons. Nevertheless, the present results provide a coarse quantitative estimate, to our knowledge unprecedented, of the different diagnostic challenges in the two settings, at a single recruiting site, and within a largely overlapping time window.

### ctDNA testing

Several targeted and non-targeted NGS approaches were applied to mitigate the poorly actionable MTB scenario emerging from tDNA testing but, quite strikingly, appending ctDNA to tDNA testing was the single most effective measure. We provide several metrics to estimate the advantage associated with ctDNA testing, but possibly the most convincing evidence comes from the simple comparison of patients with actionable/unexploited alterations identified by tDNA vs ctDNA (25 vs 14) in the entire 124 MTB patient cohort. This is even more impressive if one considers that the 14 actionable ctDNAs were contributed by a small subset of 36 ctDNA-tested patients, as compared to a far more numerous set of 104 patients tested by tDNA. Seven of these ctDNAs were from 18 patients with no available/suitable tDNA, e.g. patients with no profiling option except ctDNA, whereas the remaining 7 were from another group of 18 patients in whom ctDNA testing was aimed to confirm bulk tDNA testing, but led instead to detect ctDNA-only alterations. Unfortunately, bulk tDNA testing, and the time elapsed (weeks to months) between tDNA and ctDNA sampling do not allow to unequivocally conclude whether ctDNA-only alterations arose as a result of tumor evolution in time, space or both. Regardless, ctDNA-only alterations may be of considerable practical relevance, since they expand therapeutic opportunities in the double-tested (tDNA/ctDNA) patient group to an extent comparable to tDNA-only testing of treatment-naïve patients (66.7% vs 76.9%).

The ctDNA advantage is supported by statistical significance, but caution must be exercised since the two ctDNA-tested groups are small (n = 18 each). In addition, lack of tDNA and/or negative tDNA profiling may underlie unappreciated clinical-molecular complexity. At any rate, the ability of ctDNA to capture therapeutic opportunities is not surprising. It was noted in a study of ours on advanced HER2 breast cancer [16] and, more relevant to the present report, in a study by the Antwerp MTB. In this latter study, 173 patients (29 tested by both tDNA and ctDNA) from the local phase 1 clinical trial facility were profiled by different, mostly outsourced, NGS panels [3]. Unlike the Antwerp group, that reported a much greater frequency of actionable alterations in ctDNA than tDNA (42.4% vs 13.3%), we detected similar percentages of patients with actionable alterations (37.5% in tDNA and 38.9% in ctDNA). The only (but clinically relevant) measurable ctDNA advantage in our study was the number of actionable/unexploited alterations, e.g. the number of patients with true therapeutic opportunities (tDNA vs ctDNA, 22.7% vs 38.9%). Unfortunately, this metric was not calculated in previous studies, precluding stringent comparisons.

In summary, despite different metrics and study designs, the Antwerp study and the present report combined concordantly show that tDNA-only and tDNA-first strategies are inferior in the real-world MTB setting. Prospective, adequately powered studies and consensus MTB alteration mapping criteria are needed to determine whether tDNA testing should be replaced by ctDNA-first, ctDNA-only, or fully parallel tDNA/ctDNA testing schemes, the third approach being favored by our data. Along this line, we provide evidence for clinical response to ctDNA-guided treatment in 4 cases, all of which were distributed in the high bracket of either or both TTP and von Hoff ratios, indicative of favorable outcomes (see below).

### Tracking the patient journey from molecular diagnosis to MTB profiling

Follow-up in a small subset of 42 routine NGS cases revealed no compliance to MTB recapture in the case of outpatients (n = 12; 0%), whereas most inpatients (26/30; 86.6%) could be downstream tracked for years. Four of them were (re)considered by the MTB, leading to non-SoC treatment in three cases.

Despite the very limited sample size, these findings draw attention on at least two important novel functions that may be ‘attached’ to real-world MTBs. First, extra-mural MTB educational programs may help to offer equal opportunities to outpatients and inpatients. Second, routine NGS profiling represents a unique opportunity to create catalogues of potential therapeutic indications for re-use at later disease stages, e.g. to design drug sequences and combinations, as shown by recent clinical trials [19], and anecdotal report of one of our MTB patients [20].

Thus, while maintaining that MTBs should exclusively focus on non-SoC, tracking patient journeys effectively bridges SoC to non-SoC, and may be an important new task of real-world MTBs.

### Access to treatment

In line with most published studies [2], tDNA- and/or ctDNA-informed therapeutic indications were cumulatively detected in a minority only of the IRE MTB patients (39/124; 31%), and only a fraction of these (22/39; 56%) actually underwent MTB-recommended treatment.

The oncologist’s decision not to treat (5/39 cases) was based on careful consideration of expected toxicity and/or the chances provided by alternative treatments, and as such may not be considered a real barrier to treatment. In contrast, clinical deterioration was a major hurdle, it was noted in 8/39 (34%) patients awaiting MTB-recommended treatment, and had two components: the technical turnaround time of NGS profiling (herein 15 days) and, to a greater extent, the time required to submit an authorization dossier to local boards and the European Medicinal Agency (EMA). Since filing the dossier is subordinated to NGS reports, we strived to improve on this. From August 2022 (at the end of the present accrual period) the IRE MTB elected to enrol patients while on last standard therapy line, and not on overt progression. Hopefully, this may accelerate access to treatment.

As to cost/reimbursement, treatment was secured by the IRE MTB through institutional and public money in most cases, compassionate use and clinical trials giving minimal contributions. Clearly, real-world MTBs need more support by the national healthcare system and the pharmaceutical industry, but a key issue is the geographical distance of the site hosting applicable clinical trials, particularly for rare indications. Possibly, a substantial revision of the classical trial recruitment scheme is needed. For instance, trial inclusion should be warranted *ex-post*, even when patients are from a center/hospital where the applicable trial had not been initially activated. *Ex-post* accrual should take place with minimal administrative burden, and ideally drugs should travel to the patient/MTB site (whichever its geographical location) and not viceversa. Shared clinical trial/patient registries, open accrual schemes, unified decision support tools, and virtual MTB/trial networks (i.e. across EU) are needed. Ongoing multi-center studies, such as Can.HEAL (www.canheal.eu) may provide an efficient EU framework to speed up this process. Our data show that this strategy, once applied, may meet with success, because the patient’s decision to be treated elsewhere or withdrawal from the MTB study accounted for a small fraction (4/39; 10%) of all drop-offs.

In summary, we concur with most previous MTB studies that matching patients and drugs is often successful, but obtaining drugs is a major hindrance [21], and provide a rough estimate of the relative impact of factors hampering access to MTB-recommended treatment in Italy, and possibly EU.

### Outcome

Population (TTP) and patient-specific (Von Hoff ratio) outcome metrics were applied to evaluate MTB-recommended treatments. TTP ranged from 2 to 30 months approximately, 9/19 patients exceeded the 1.3 Von Hoff efficacy threshold, and two of three patients with the longest TTP also had high von Hoff ratios. However, neither TTP nor the Von Hoff ratio correlated with demographics, best response, and number or duration of pre-MTB therapy lines. Given the clinical-pathological heterogeneity of the MTB patient cohort, this is far from surprising. Also, presumably due to its limited numerosity, our study failed to detect a correlation between actionability level and outcome, that was instead detected by a large clinical study (n = 516 patients) steered by the Gustav Roussy MTB [12].

While looking for a metric associated with outcome, we noted that further treatment had indeed been administered (as per physician’s choice) to a subset (8/19) of MTB patients after progression on MTB-recommended therapy. Post-MTB treatment (one to three lines) had altogether a duration comparable to that of MTB-recommended treatment, which is in itself remarkable. Multiple variables define a more favourable clinical profile of patients receiving post-MTB treatment as compared to patients who had received no post-MTB treatment. This favorable profile included a significantly greater number of pre-MTB therapy lines, a trend for longer duration of these standard treatments, a significantly longer TTP of MTB-recommended therapy, and a much longer overall survival calculated from the time of MTB accrual. In addition, duration of MTB and post-MTB treatments correlated in these 8 patients, which provides evidence for individual outcome-associated factors. It then appears that a subset of patients/tumors is inadvertently selected displaying an elective susceptibility to diverse/multiple treatments.

## Conclusions

In this report, we tentatively list accrual and alteration mapping criteria distinguishing real-world MTBs from other clinical multidisciplinary organisms and molecular oncology boards. By applying these criteria, we show that real-world MTBs will considerably benefit from more intensive ctDNA testing, and a downstream patient tracking system connecting routine NGS with the MTB setting. It is also noted that MTB patients need better access to clinical trials through a complete reorganization of accrual and drug supply chains (open access to drugs within real-world MTB networks). Although we report remarkable objective responses to non-SoC treatment, MTB patients are a very special case, and extrapolation of MTB results to the general cancer population should correct for inadvertent and systematic selection of patients electively susceptible to diverse/multiple treatments.

### Supplementary Information

**Additional file 1: Table S1.**a MTB patients and actionable alterations by tumor histotype. b. Routine NGS patients and actionable alterations by tumor histotype. c. MTB vs routine NGS patients: distribution of actionable alterations in tumor histotypes common to the two populations. **Table S2.** MTB genomic profiling: ctDNA-only alterations. **Table S3.** MTB patients: OncoKB levels and objective response rates.

## Data Availability

The datasets used and/or analysed during the current study are stored in-house in a pseudo-anonymized format, in the dedicated MTBO platform. Access is available from the corresponding author on reasonable request.

## Abbreviations

MTB: Molecular tumor board
tDNA: Tumor DNA
ctDNA: Circulating tumor DNA
SoC: Standard of care
non-SoC: Non-standard of care
ESMO: European society of medical oncology
PS: Performance status
NGS: Next generation sequencing
EMA: European medicines agency
AIFA: Agenzia Italiana del Farmaco
WES: Whole exome sequencing
WTS: Whole transcriptome sequencing
VEP: Variant effect predictor
PCGR: Personal cancer genome reporter
RT-PCR: Reverse transcription PCR
dPCR: Digital PCR
MTBO: Molecular tumor board orchestrator
SD: Standard deviation (SD)
95%CI: 95% Confidence intervals
TTP: Time to progression
OR: Odd ratio
