# Supplementary material for: The Molecular Tumor Board of the Regina Elena National Cancer Institute: from accrual to treatment in real-world
Source: J Transl Med. 2023 Oct 16;21:725. doi: 10.1186/s12967-023-04595-5 (PMC10577953; doi:10.1186/s12967-023-04595-5)
Supplement: Supplementary file 1 — Additional file 1: Table S1.a MTB patients and actionable alterations by tumor histotype. b. Routine NGS patients and actionable alterations by tumor histotype. c. MTB vs routine NGS patients: distribution of actionable alterations in tumor histotypes common to the two populations. Table S2. MTB genomic profiling: ctDNA-only alterations. Table S3. MTB patients: OncoKB levels and objective response rates. [file 12967_2023_4595_MOESM1_ESM.docx]

***Table S1a***

***MTB patients and actionable alterations by tumor histotype***

|  |  | |  | | |  |  | |  |
| --- | --- | --- | --- | --- | --- | --- | --- | --- | --- |
| **MTB (126 pts)** | | | | | | | | | |
| Type of cancer | | *# pts* | | *# pts with actionable alterations* | *% pts with actionable alterations (tumor type)* | | | *% pts with actionable alterations (overall)* |  |
|  | |  | |  |  | | |  |  |
| breast carcinoma | | 20 | | 10 | 50,0% | | | 20,8% |  |
| NSCLC | | 18 | | 14 | 77, 8% | | | 29,2% |  |
| colorectal carcinoma | | 17 | | 4 | 23, % | | | 8,3% |  |
| brain cancer | | 12 | | 6 | 50,0 % | | | 12,5% |  |
| sarcoma | | 11 | | 3 | 27,3% | | | 6,2% |  |
| melanoma | | 10 | | 6 | 60,0% | | | 12,5% |  |
| multiple cancers | | 7 | | 0 | 0,0% | | | 0,0% |  |
| pancreatic cancer | | 7 | | 1 | 14,3% | | | 2,1% |  |
| ovarian cancer | | 6 | | 1 | 16,7% | | | 2,1% |  |
| gastric cancer | | 3 | | 0 | 0,0% | | | 0,0% |  |
| renal carcinoma | | 2 | | 0 | 0,0% | | | 0,0% |  |
| leukemia | | 2 | | 1 | 50,0% | | | 2,1% |  |
| medullary thyroid cancer | | 2 | | 2 | 100,0% | | | 4,2% |  |
| salivary gland carcinoma | | 2 | | 0 | 0,0% | | | 0,0% |  |
| biliary carcinoma | | 1 | | 0 | 0,0% | | | 0,0% |  |
| cholangiocarcinoma | | 1 | | 0 | 0,0% | | | 0,0% |  |
| larynx carcinoma | | 1 | | 0 | 0,0% | | | 0,0% |  |
| neuroblastoma | | 1 | | 0 | 0,0% | | | 0,0% |  |
| paraganglioma | | 1 | | 0 | 0,0% | | | 0,0% |  |

***Table S1b***

***Routine NGS patients and actionable alterations by tumor histotype***

| **Routine NGS (147 pts)** | | | | | | | | | | |  |
| --- | --- | --- | --- | --- | --- | --- | --- | --- | --- | --- | --- |
| *Type of cancer* | | | *# pts* | *# pts with actionable alterations* | | *% pts with actionable alterations (tumor type)* | | *% pts with actionable alterations (overall)* |  | |  |
|  | | |  |  | |  | |  |  | |  |
| lung cancer | | | 75 | 70 | | 93,3% | | 61,9% |  | |  |
| colorectal carcinoma | | | 45 | 28 | | 62,2% | | 24,8% |  | |  |
| brain cancer | | | 12 | 6 | | 50,0% | | 5,3% |  | |  |
| cholangiocarcinoma | | | 5 | 4 | | 80,0% | | 3,5% |  | |  |
| GIST | | | 3 | 0 | | 0,0% | | 0,0% |  | |  |
| ovarian cancer | | | 2 | 2 | | 100,0% | | 1,8% |  | |  |
| salivary gland carcinoma | | | 2 | 2 | | 100,0% | | 1,8% |  | |  |
| breast cancer | | | 1 | 0 | | 0,0% | | 0,0% |  | |  |
| gastric Cancer | | | 1 | 0 | | 0,0% | | 0,0% |  | |  |
| prostate cancer | | | 1 | 1 | | 100,0% | | 0,9% |  | |  |
|  |  |  | | |  | |  | | |  | |

***Table S1c***

***MTB vs routine NGS patients: distribution of actionable alterations in tumor histotypes common to the two populations***

|  | *patient populations* | |
| --- | --- | --- |
|  | *MTB* | *Routine NGS* |
| Type of cancer | *n=35* | *n=112* |
| lung cancer | 14 (40,0%)* | 70 (62,5%)* |
| colorectal carcinoma | 4 (11,4%) | 28 (25,0%) |
| brain cancer | 6 (17,1%) | 6 (5,4%) |
| cholangiocarcinoma | 0 (0,0%) | 4 (3,6%) |
| ovarian cancer | 1 (2,9%) | 2 (1,8%) |
| salivary gland carcinoma | 0 (0,0%) | 2 (1,8%) |
| breast cancer | 10 (28,6%) | 0 (0,0%) |

* n pts (per cent with actionable alterations)

c^2^ test (all alterations, MTB vs routine NGS) p <0,001

**Table S2**

**MTB genomic profiling: ctDNA-only alterations**

| pt # | cancer | alteration | OncoKB level* | tDNA testing | ctDNA testing |
| --- | --- | --- | --- | --- | --- |
|  |  |  |  |  |  |
| 24 | Breast | ESR1 p.Y537C | 3A | Focus | PanCancer, dPCR |
| 29 | Breast | ESR1 Y537S/N | 3A/B | CHPV2 | PanCancer |
| 39 | Breast | PIK3CA p.E545K | 3A | CHPv2 | PanCancer, dPCR |
| 15 | NSCLC | EGFR p.L858R | 1 | CHPv2 | PanCancer, dPCR |
| 80 | NSCLC | EGFR E746_A750del15 | 3A | CHPv2 | F-One liquid |
| 101 | NSCLC | RET/CCDC6 fusion | 1 | Focus | PanCancer |
| 104 | NSCLC | RET/CCDC6 fusion | 1 | OPA | PanCancer |

*at the time of recruitment

**Table S3**

**MTB patients: OncoKB levels and objective response rates**

|  | **PD**  **N=4** | **SD/PR**  **N=15** | **Fisher’s exact test**  ***p-value*** |
| --- | --- | --- | --- |
| **Ono KB 1/2** | 1 (14.3) | 6 (85.7) | 0.999 |
| **OncoKB 3A/B** | 3 (25.0) | 9 (75.0) |  |
|  |  |  |  |
